# Determinants of Quality of Life in High-Dose Benzodiazepine Misusers

**DOI:** 10.3390/ijerph14010038

**Published:** 2017-01-04

**Authors:** Stefano Tamburin, Angela Federico, Marco Faccini, Rebecca Casari, Laura Morbioli, Valentina Sartore, Antonio Mirijello, Giovanni Addolorato, Fabio Lugoboni

**Affiliations:** 1 Department of Neurosciences, Biomedicine and Movement Sciences, University of Verona, Piazzale Scuro 10, Verona 37134, Italy; angela.federicovr@gmail.com; 2 Department of Internal Medicine, Addiction Unit, Verona University Hospital, Piazzale Scuro 10, Verona 37134, Italy; marco.faccini@ospedaleuniverona.it (M.F.); rebecca.casari@ospedaleuniverona.it (R.C.); laura.morbioli@gmail.com (L.M.); valentina.sartore@studenti.univr.it (V.S.); fabio.lugoboni@ospedaleuniverona.it (F.L.); 3 Alcohol Use Disorders Unit, Department of Internal Medicine, Gastroenterology and Hepatology, Catholic University of Rome, Rome 00168, Italy; antonio.mirijello@gmail.com (A.M.); giovanni.addolorato@unicatt.it (G.A.)

**Keywords:** 12-Items General Health Questionnaire (GHQ-12), addiction, benzodiazepine (BZD), drug misuse, multivariable analysis, patient-centered outcomes, quality of life (QoL), Short Form-36 (SF-36)

## Abstract

Benzodiazepines (BZDs) are among the most widely prescribed drugs in developed countries, but they have a high potential for tolerance, dependence and misuse. High-dose BZD misuse represents an emerging addiction phenomenon, but data on quality of life (QoL) in high-dose BZD misusers are scant. This study aimed to explore QoL in high-dose BZD misuse. We recruited 267 high-dose BZD misusers, compared the QoL scores in those who took BZD only to poly-drug misusers, and explored the role of demographic and clinical covariates through multivariable analysis. Our data confirmed worse QoL in high-dose BZD misusers and showed that (a) QoL scores were not negatively influenced by the misuse of alcohol or other drugs, or by coexisting psychiatric disorders; (b) demographic variables turned out to be the most significant predictors of QoL scores; (c) BZD intake significantly and negatively influenced QoL. Physical and psychological dimensions of QoL are significantly lower in high-dose BZD misusers with no significant effect of comorbidities. Our data suggest that the main reason for poor QoL in these patients is high-dose BZD intake per se. QoL should be considered among outcome measures in these patients.

## 1. Introduction

Benzodiazepines (BZDs) and BZD-related drugs represent a group of gamma-aminobutyric acid (GABA)-ergic molecules, which are extensively prescribed for a wide range of indications, and they are one of the most widely used groups of pharmaceuticals worldwide [1]. BZDs are indicated for the management of anxiety and insomnia, but a short-term prescription is recommended because of their high potential for tolerance and dependence [2]. Epidemiological figures indicate that 2%–7.5% of the general population in developed countries [3] and 6%–76% of patients on BZDs become long-term (i.e., greater than six months) users [2]. The low toxicity coupled with a high potential for tolerance raises the risk that patients on BZDs increase their daily dosage [4]. It is reported that 20%–50% of patients on BZDs report some sort of withdrawal when trying discontinuation, and 3%–4% of them show clear signs of misuse or dependence [1]. Surveys from France, Germany, Italy and the UK suggest that 3.2%–3.9% of BZD users exceed the recommended dose [5], and it is estimated that approximately 0.16% of the Swiss adult population are high-dose BZD misusers [6]. High-dose BZD misuse represents an emerging addiction phenomenon, and it is defined as BZD intake for more than six months, a dose equivalent >50 mg diazepam/day, and/or an otherwise problematic use of BZDs, such as mixing BZDs, escalating dosage, using BZDs for recreational purposes, or obtaining BZDs illegally [7,8].

Quality of life (QoL) is a complex concept that is defined as “an individual’s perception of his/her position in life, in the context of culture and value systems, in which he/she lives and in relation to his/her goals, expectations, standards, and concerns” [9]. QoL has increasingly been considered as a prerequisite for the overall health of people, including satisfaction, happiness and well-being. Converging evidence indicates that addiction or misuse of many drugs, including alcohol, opioids, and cocaine, may affect QoL [10,11,12,13,14,15]. Data on QoL of BZD misusers mainly come from poly-drug misusers [13,16,17,18] and/or patients with psychiatric comorbidities [19,20], making the understanding of the specific impact of BZD misuse difficult. A recent study from our group showed lower QoL and social functioning, along with high levels of psychological distress, in a small group of high-dose BZD misusers without alcohol or drug co-dependence [8]. However, high-dose BZD misusers frequently show coexisting psychiatric conditions and/or other drug dependence or misuse, and these factors may influence QoL together with demographic ones (e.g., age, sex/gender). Whether the interplay of these factors has an effect on QoL of high-dose BZD misusers has never been explored.

The goal of the present study was to add new pieces of information on QoL in high-dose BZD misuse. To this aim, we recruited a large group of high-dose BZD misusers, compared the QoL in patients who misused BZD only (either one or more BZD active principles) to poly-drug misusers (i.e., BZD plus alcohol or other drugs), and explored the role of demographic and clinical covariates on QoL scores.

## 2. Subjects and Methods

### 2.1. Patients

We recruited 267 patients (age > 18 years), who were consecutively seen from May 2013 to May 2016 at the Department of Internal Medicine, Addiction Unit, Verona University Hospital, Italy, for high-dose BZD misuse, which was defined as a diagnosis of BZD dependence according to the Diagnostic and Statistical Manual of Mental Disorders Fourth Edition (DSM)-IV criteria [21] with misuse lasting more than six months, and daily BZD intake exceeding at least five times the maximum daily recommended dose (i.e., >50 mg diazepam/day) [7,8]. In the present study, psychiatric disorders and addiction to alcohol or other drugs, even if in remission, were not considered as exclusion criteria, because we were interested in their role as cofactors in influencing QoL. 

The dosage of BZDs was standardized as the daily diazepam dose equivalent (DDDE, mg) according to previous studies [8]. Zolpidem, which is an imidazopyridine compound and is chemically distinct from BZDs, has been included among BZDs in the present study, since it binds to the alpha-1 subtype of the BZD receptor and its effects are similar to those of BZDs [22].

Demographic (sex; age; education: grade school, high school, university; employment: unemployed, employed; marital status: single or divorced, engaged or married), and clinical variables (type of drug misuse: BZD only either one or more active principles, active poly-drug misuse defined as BZD plus alcohol or other drugs, previous poly-drug misuse; other drugs of misuse: alcohol, opioids, cocaine, cannabinoids, barbiturates; DDDE: mg; BZD misuse duration: months; presence and type of coexisting major psychiatric diseases excluding anxiety disorders and mild depression: major depression, other psychoses, personality disorders) were recorded. Information on these variables was mainly obtained from medical records. DDDE data were based on self-report. For prior vs. active poly-drugs misuse, a time frame of 12 months from the time of enrollment was chosen.

The study was conducted according to the Declaration of Helsinki and approved by the ethics committee of the Verona University Hospital (study protocol number 875CESC). All patients gave informed consent for participation to the study.

### 2.2. Quality of Life Measures

All the patients sat in a quiet room without any disturbing factors and completed two questionnaires, i.e., the Short Form-36 (SF-36) questionnaire and the 12-item General Health Questionnaire (GHQ-12). 

The SF-36 is a generic QoL scale consisting of 36 individual items that are grouped into eight dimensions: physical functioning (PF), role physical (RF), bodily pain (BP), general health (GH), vitality (VT), social functioning (SF), role emotional (RE), mental health (MH), with a score from 0 (worst score) to 100 (best score) for each dimension [23,24]. 

The GHQ-12 is one of the most widely used screening tool to identify short-term changes in psychological health and is composed of 12 questions on mood states over the previous two weeks: lost sleep, feelings of being under strain, could not concentrate, felt unable to play a useful role, could not face problems, could not make decisions, could not overcome difficulties, felt unhappy, did not enjoy day-to-day activities, felt depressed, lost confidence, and felt worthless [25]. GHQ-12 answers were scored on a two-point scale (coded 0-0-1-1), resulting in 0–12 total score range with higher values indicating more severe psychological distress [26], and a cut-off value of ≥4 [8]. SF-36 and GHQ-12 were administered prior to detoxification with flumazenil infusion [22].

### 2.3. Statistical Analysis

All tests were carried with the IBM SPSS version 20.0 statistical package. The Fisher’s exact and the Pearson’s χ^2^ test were used for categorical variables, while the one-way ANOVA and post-hoc with Bonferroni’s correction were used for continuous variables in case of normal distribution, otherwise the non-parametric Mann-Whitney U and Kruskal Wallis tests were applied. Multivariable analysis was used to explore the influence of the demographic and clinical covariates (sex, age, education, employment, marital status, type of drug misuse, type of poly-drug misuse, DDDE, BZD misuse duration, presence and type of coexisting major psychiatric diseases) on QoL measures. Variables that were unique to poly-drug misusers (e.g., alcohol) were set as 0 in BZD only misusers. Linear regression model analysis was applied for SF-36 dimensions (continuous dependent variables). Logistic regression model analysis was used for GHQ-12 (binary dependent variable: ≥4, <4), and the results were expressed as odd ratios (ORs) and 95% confidence intervals (CI). The goodness of fit of the logistic regression model was assessed using the Hosmer and Lemeshow test [27]. *p* < 0.05 (two-tailed) was taken as the significance threshold for all the tests.

## 3. Results

### 3.1. Patients

In our sample, 166 patients misused only BZDs (either one or more BZD active principles), while 49 were active poly-drug misusers excluding tobacco and 52 were previous poly-drug misusers in remission at the time of recruitment. 

Among demographic variables, only sex and age significantly differed according to the type of BZD misuse (Table 1). Post-hoc showed a significant age difference for the comparison between BZD only and active poly-drug misusers.

All 267 patients misused at least one BZD, 39 of them misused two BZDs, and four misused three different BZDs. The types and frequencies of BZDs (main active principle in case of more than one BZD) and other drugs of misuse are reported in Table 2. The second most misused BZDs were alprazolam (*n* = 10), lormetazepam (*n* = 4), triazolam (*n* = 4), lorazepam (*n* = 3), bromazepam (*n* = 2), zolpidem (*n* = 1), other (*n* = 15), while the third most misused BZDs were lormetazepam (*n* = 1), alprazolam (*n* = 1), zolpidem (*n* = 1), other (*n* = 1). The DDDE ranged from 55 to 2330 mg (median = 250 mg). The type of BZD differed according to active poly-drug abuse, but no difference in DDDE (mg) and BZD misuse duration (months) was found when comparing BZD only vs. poly-drug misusers (Table 2). 

Coexistent major psychiatric diseases (other psychoses and personality disorders) were significantly more frequent in patients with active poly-drug misuse than those who misused BZDs only (Table 2).

### 3.2. Quality of Life Measures

**Figure 1:**
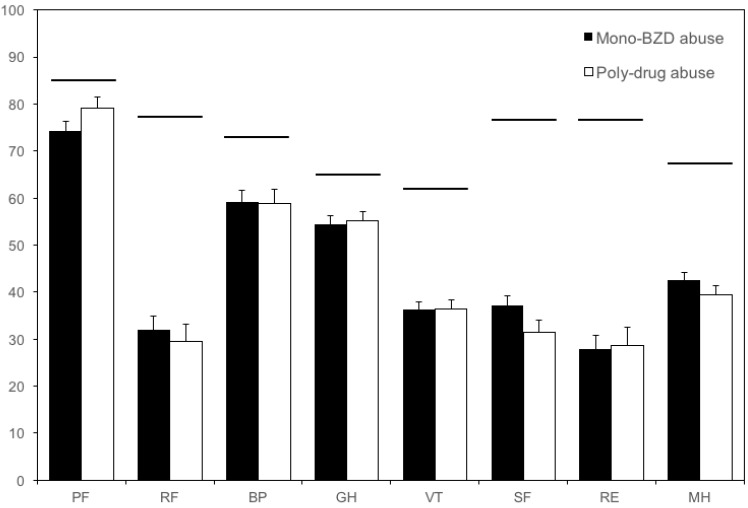
Score in the SF-36 dimensions. Closed bars: mono-BZD misusers (either one or more BZDs, *n* = 166); open bars: poly-drug misusers (*n* = 101). The SF-36 scores ranged from 0 (worst score) to 100 (best score). None of the dimensions significantly differed between the two groups. Horizontal bars indicate the mean score in the Italian population [8,24]. Vertical error bars equal one SEM. BP: Bodily pain; BZD: Benzodiazepine; GH: General health; MH: Mental health; PF: Physical functioning; RE: Role emotional; RF: Role physical; SF: Social functioning; SF-36: Short Form-36; VT: Vitality.

None of the SF-36 dimensions was significantly different when comparing patients who misused BZD only vs. poly-drug misusers, either previous or active (Mann-Whitney U test: n.s. for all dimensions; Figure 1).

The comparison was repeated by exploring active BZD misuse (i.e., BZD only misuse + previous poly-drug misuse; *n* = 218) vs. active poly-drug misuse (*n* = 49), but again none of the SF-36 dimensions turned out to be significantly different between the two groups (Mann-Whitney U test).

By using the cut-off value of ≥4 for the GHQ-12 score, 122 out of the 166 patients who misused BZD only (73.5%) and 79 of the 101 either previous or active poly-drug misusers (78.2%) showed severe psychological distress (Fisher’s exact test: n.s.).

The severe psychological distress was slightly higher in the 218 active BZD misusers (81.6%) than in the 49 active poly-drug misusers (70.2%), but the difference did not reach statistical significance (Fisher’s exact test: n.s.).

The multivariable linear regression model was applied to explore the influence of demographic and clinical covariates on SF-36 scores. Among demographic variables, age significantly influenced all SF-36 dimension scores (i.e., higher scores in older patients but to a variable extent according to the SF-36 dimension), while sex (i.e., higher BP, VT and MH scores in males), education (i.e., higher PF, BP, GH, VT and MH scores in patients with higher education), employment (i.e., higher PF and GH scores in employed patients), and marital status (i.e., higher PF, VT and MH scores in engaged or married patients) were found to significantly influence some SF-36 scores, but these findings were not consistent across all SF-36 dimensions (Table 3).

Among clinical variables, only poly-drug misuse, either active or previous, was found to significantly influence PF (i.e., higher PF score in patients with poly-drug misuse than in those on BZDs only), and DDDE significantly influenced SF (i.e., higher SF score in patients who took less DDDE), but the other clinical variables, including active poly-drug misuse, did not significantly influence SF-36 scores (Table 3).

The multivariable logistic regression model showed that each unitary increment of DDDE (mg) significantly influenced the risk of a GHQ-12 score ≥4 (OR = 1.01, 95% CI: 1.00–1.02, *p* = 0.012), while the remaining covariates were not significant.

## 4. Discussion

Our data confirmed worse QoL in high-dose BZD misusers in comparison to that expected in the reference Italian population [8,24], and offered these new findings, which will be discussed below: (a) SF-36 scores and the percentage of patients with a GHQ-12 score ≥4 were not significantly influenced by the misuse of alcohol or other drugs; (b) poly-drug misuse resulted in a higher PF score in comparison to misuse of BZD only; (c) coexisting psychiatric disorders did not influence QoL outcomes; (d) demographic variables turned out to be the most significant predictors of SF-36 scores; (e) BZD intake expressed as DDDE significantly and negatively influenced SF and the risk of a GHQ-12 score ≥4.

Patients’ self-reported outcomes have become an increasingly important source of information in health care [11]. QoL measures may provide insight into a broader perspective on physical, mental and social health [11,28], and may help to gradually shift the clinical focus from a cure to the enhancement of this outcome [29]. This view fits well with the recognition that substance misuse is a chronic, relapsing disorder that may have negative consequences on various life domains [29,30,31,32]. Early QoL studies among patients with substance use disorders did not allow general conclusions due to the small number of studies, the small samples, and the use of different measures (i.e., health-related QoL, general QoL) and tools [31], but more recent reports offered more insight on this topic [29].

All SF-36 dimensions were worse than those in the general population in the present study. This finding confirmed that from a previous report from our group in a small group of high-dose BZD patients seeking detoxification, without either poly-drug misuse or mental disorders [8]. The present results offer a broader view on this topic, because the sample size was larger, and we included patients with coexisting psychiatric conditions and/or other drug dependence or misuse, offering a more real-life scenario and allowing the exploration of the influence of each single variable on QoL scores through multivariable analysis.

Data from other substance use disorders indicate an overall reduction of QoL [29]. Despite studies investigating QoL in opiate-dependent individuals used different tools, a systematic review and recent data indicate that these patients show lower QoL scores compared with the general population and people with various medical illnesses [9,11], and that methadone treatment can ameliorate QoL [33]. General and disease-specific QoL measures are lower in patients with alcohol misuse and dependence, but may be improved by treatment and successful abstinence [29,34]. Some reports did not detect any impact of cocaine use on QoL [29], while other ones demonstrated QoL impairment in the initial phase of drug dependence, and a correlation between the severity of cocaine dependence and QoL [10]. Data on nicotine misuse are more contradictory. While ex-smokers appear to have higher QoL scores in comparison to current smokers [29,35], patients attempting to quit smoking show a decrease in QoL [36]. It is conceivable that the reason for worse QoL in patients with substance use disorders may include the disruption of normal daily life activities and social contacts in relation to the time and money required to obtain the drug of misuse, and the physical, emotional and cognitive side effects of the drug [8].

Quite surprisingly, the presence of poly-drug misuse did not lower QoL scores in our sample. Interestingly, any poly-drug misuse, i.e., either active or previous, resulted in a higher SF-36 PF than the misuse of BZD only. Furthermore, active poly-drug misuse was not found to influence (i.e., either significantly reduce or increase) any of the QoL scores in multivariable analysis. Our findings seem to be in contrast with the previous literature, which showed that the concurrent dependence or misuse of two substances is associated with worse QoL [37]. We may speculate that, in our patients, high-dose BZD misuse resulted in such a severe reduction of QoL measures that the concurrent substance use disorder could not cause a lower score of this outcome because of a ceiling effect. Our hypothesis is in keeping with recent data showing that QoL in high-dose BZD misuse was worse than in heroin-addicted patients, despite the latter ones being treated by methadone [38].

Previous studies documented a negative effect of mental disorders on QoL [39,40]. Psychiatric comorbidity was not found to significantly influence QoL measures in our patients. This is in keeping with data from opiate dependence, where no difference was found between QoL scores in patients with and those without psychiatric disorders [41]. In contrast, a recent report showed that patients with substance use disorders (alcohol, cannabis, cocaine) and psychiatric comorbidity evaluated their QoL more negatively than those without psychiatric comorbidity [42]. It is difficult, however, to disentangle the directionality of the association between mental health issues and drug misuse and dependence in substance use disorders, because patients with psychiatric diseases may misuse substances they have been prescribed, and mental health problems may be secondary to drug misuse [34]. In our study, anxiety and mild depression were not considered as psychiatric comorbidities because these two conditions are very common in high-dose BZD misuse, but they may play a role in worsening QoL.

In accordance with previous studies on QoL in patients with substance use disorders, demographic variables that resulted in worse QoL scores in our patients were female sex, unemployment, and being single or divorced [8,9,34]. While the negative effect of being unemployed and single/divorced can be easily understood, the reason for worse QoL in women may stem from the significantly higher prevalence of anxiety and depression in comparison to men [43].

We found a significant positive effect of age on most SF-36 dimensions in our cohort of patients. This finding is difficult to compare with previous studies, because of the lack of data on the effect of age on QoL in other substance use disorders. Age was found to significantly influence QoL, either positively or negatively, in normal controls, with a prevalent negative effect because of aging-related problems [44]. We may speculate that the young-to-middle age of our sample could have mitigated the potentially negative influence of physical problems secondary to aging and their limitations on daily activities and physical functioning [44]. Future studies should confirm this hypothesis.

We documented that BZD intake, expressed as DDDE, significantly reduced the SF score and enhanced the risk of a GHQ-12 score ≥4. Data from low-dose BZD users showed that initial BZD intake caused improvement in QoL, likely because of anxiety reduction without consistent side effects [45]. Taken together, these data suggest that limited duration of use and low BZD dosage, as suggested by guidelines [1], may improve QoL, while chronic misuse and very high doses, as in our sample, consistently reduce physical and emotional QoL dimensions.

A limitation of the present study is that DDDE was based on patients’ self-reporting and the presence of alcohol and other drug co-misuse was not confirmed by urine toxicology. Another limitation is the cross-sectional design and the absence of a follow-up to evaluate QoL changes in response to treatment. Other limitations include the sample of convenience from a single treatment hospital, the limited availability of covariates representing clinical characteristics, the absence of correction for multiple comparisons, and the generalizability of results to low-dose BZD users or those not in treatment for high-dose BZD misuse. Evidence from low-dose BZD [18] and other drugs of misuse (e.g., opioids, alcohol) [11,33,34] indicates that treatment results in improved QoL. Future studies should follow up with patients after successful treatment for BZD misuse to better explore the specific contribution to QoL of high-dose BZDs, and whether QoL might represent a relevant outcome measure in this field or a prognostic factor for detoxification-seeking or drug misuse relapse [46].

## 5. Conclusions

In conclusion, we confirmed that physical and psychological dimensions of QoL were significantly lower in high-dose BZD misusers, and that comorbidities (i.e., poly-drug misuse, psychiatric diseases) did not influence QoL scores. These data suggest that the main reason for poor QoL in these patients is high-dose BZD intake per se. Since the administration of a QoL instrument takes roughly 10–15 min, expanding our knowledge on areas of deficit would enable more focused interventions aimed to address specific QoL dimensions [34].

## Figures and Tables

**Table 1:** Demographic characteristics of the patients according to the type of high-dose benzodiazepines (BZD) and poly-drug misuse.

Variable	Type of BZD Misuse			*p* Value
	Only BZD Misuse (*n* = 166)	Previous Poly-Drug Misuse (*n* = 52)	Active Poly-Drug Misuse (*n* = 49)	
Sex (M, F)	33%, 67%	82%, 18%	67%, 33%	<0.001 *
Age	45.7 ± 10.7	44.2 ± 8.9	41.4 ± 9.0	0.03 *
Education ^†^	23%, 51%, 26%	33%, 54%, 13%	31%, 53%, 16%	n.s.
Employment ^‡^	37%, 63%	48%, 52%	45%, 55%	n.s.
Marital status ^§^	54%, 46%	69%, 31%	61%, 39%	n.s.

M: Male. F: Female. n.s.: Not significant. ^†^ Education: grade school, high school, university (%). ^‡^ Employment: unemployed, employed (%). ^§^ Marital status: single/divorced, engaged/married (%). * Significant statistical comparison (*p* < 0.05).

**Table 2:** Type of BZD misused by patients, daily dosage, misuse duration, other drugs of misuse and coexisting psychiatric conditions.

Variable	Type of BZD Misuse			*p* Value
	All Patients (*n* = 267)	Active BZD ^^^ (*n* = 218)	Active Poly-Drug (*n* = 49)	
Active principle ^†^				0.015 *
Lormetazepam	187 (70.0%)	156 (71.6%)	31 (63.3%)	
Alprazolam	22 (8.2%)	17 (7.8%)	5 (10.2%)	
Zolpidem	22 (8.2%)	20 (9.2%)	2 (4.1%)	
Lorazepam	12 (4.5%)	6 (2.8%)	6 (12.2%)	
Bromazepam	10 (3.7%)	7 (3.2%)	3 (6.1%)	
Triazolam	4 (1.5%)	2 (0.9%)	2 (4.1%)	
Other BZDs	10 (3.7%)	10 (4.5%)	0 (0.0%)	
DDDE (mg) ^‡^	394.5 ± 392.0	406.2 ± 401.1	365.9 ± 324.5	n.s.
Misuse duration (mos)	74.9 ± 69.8	75.7 ± 72.1	71.4 ± 58.3	n.s.
Other drugs of misuse ^§^				n.a.
Alcohol	39/33/29	25/27/0	14/6/29	
Opioids	70/26/5	37/15/0	33/11/5	
Cocaine	47/43/11	29/23/0	18/20/11	
Cannabinoids	57/35/9	32/20/0	25/15/9	
Barbiturates	96/3/1	50/1/0	46/2/1	
Psychiatric diseases ^¶^	164 (61.4%)	125 (57.3%)	39 (79.6%)	0.004 *
Major depression	145 (54.3%)	125 (45.9%)	20 (40.8%)	n.s.
Other psychoses	28 (10.5%)	15 (11.7%)	13 (26.5%)	<0.001 *
Personality disorders	22 (8.2%)	13 (6.0%)	9 (18.4%)	0.009 *

DDDE: Daily diazepam dose equivalent. mos: Months. n.s.: Not significant. n.a.: Not applicable. ^†^ Main BZD in case of misuse of different BZDs. ^‡^ Sum of all the DDDEs in case of poly BZD misuse. ^§^ Other drugs of misuse: No/previous/active. ^¶^ Psychiatric diseases: Coexisting major conditions excluding anxiety disorders and mild depression. ^^^ Active BZD: BZD only misuse + Previous poly-drug misuse. * Significant statistical comparison (*p* < 0.05).

**Table 3:** Linear regression model analysis for the SF-36 dimensions.

SF-36 Dimensions and Significant Covariates	β	95% CI	*p* Value
Physical functioning (PF)			
Age (year)	1.19	1.01; 1.37	<0.001
Education	8.96	3.81; 14.12	0.001
Employment	10.94	3.70; 18.18	0.003
Marital status	8.53	0.84; 16.22	0.030
Poly-drug misuse (either active or previous)	14.69	7.58; 21.80	<0.001
Role physical (RF)			
Age (year)	0.68	0.58; 0.79	<0.001
Bodily pain (BP)			
Sex	−9.66	−17.70; −1.60	0.019
Age (year)	1.10	0.92; 1.28	<0.001
Education	9.98	4.18; 15.79	0.001
General health (GH)			
Age (year)	0.90	0.75; 1.04	<0.001
Education	5.61	1.37; 9.84	0.010
Employment	7.06	1.18; 12.94	0.019
Vitality (VT)			
Sex	−9.55	−14.70; −4.40	<0.001
Age (year)	0.87	0.75; 0.99	<0.001
Education	4.32	0.78; 7.87	0.017
Marital status	6.99	1.48; 12.50	0.013
Social functioning (SF)			
Age (year)	0.83	0.65; 1.01	<0.001
DDDE (mg)	−0.02	−0.01; −0.04	0.026
Role emotional (RE)			
Age (year)	0.71	0.56; 0.87	<0.001
Mental health (MH)			
Sex	−7.39	−12.95; −1.83	0.009
Age (year)	0.95	0.82; 1.08	<0.001
Education	4.92	1.09; 8.75	0.012
Marital status	7.38	1.43; 13.32	0.015

Here are reported only covariates that turned out to be significant in multivariable linear regression analysis. Please note that higher SF-36 scores indicated higher quality of life levels. Sex: 0 = male, 1 = female; education: 0 = grade school, 1 = high school, 2 = university; employment: 0 = unemployed, 1 = employed; marital status: 0 = single or divorced, 1 = engaged or married; poly-drug misuse: 0 = no, 1 = yes. DDDE: Daily diazepam dose equivalent. SF-36: Short Form-36.
